# An immunotherapy survivor population: health-related quality of life and toxicity in patients with metastatic melanoma treated with immune checkpoint inhibitors

**DOI:** 10.1007/s00520-019-04818-w

**Published:** 2019-05-14

**Authors:** Aine O’Reilly, Peta Hughes, Jasmine Mann, Zhuangming Lai, Jhia Jiat Teh, Emma Mclean, Kim Edmonds, Karla Lingard, Dharmisha Chauhan, Joanna Lynch, Lewis Au, Aileen Ludlow, Natalie Pattison, Theresa Wiseman, Samra Turajlic, Martin Gore, James Larkin, Olga Husson

**Affiliations:** 1 Renal and Skin Units, The Royal Marsden Hospital National Health Service Foundation Trust, London, UK; 2 Imperial College London, London, UK; 3 University of Hertfordshire/East & North Herts NHS Trust, London, UK; 4 Health Service Research, Royal Marsden Hospital National Health Service Foundation Trust, London, UK; 5 Translational Cancer Therapeutics Laboratory, The Francis Crick Institute, London, UK; 6 Division of Clinical Studies, Institute of Cancer Research, London, UK

**Keywords:** Skin cancer, Melanoma, Health-related quality of life, Immune checkpoint inhibitors

## Abstract

**Purpose:**

The immune checkpoint inhibitors (ICIs) have resulted in subgroups of patients with metastatic melanoma achieving high-quality durable responses. Metastatic melanoma survivors are a new population in the era of cancer survivorship. The aim of this study was to evaluate metastatic melanoma survivors in terms of health-related quality of life (HRQoL), immune-related adverse events (irAEs) and exposure to immunosuppressive agents in a large single centre in the UK.

**Methods:**

We defined the survivor population as patients with a diagnosis of metastatic melanoma who achieved a durable response to an ICI and had been followed-up for a minimum of 12 months from initiation of ICI without disease progression. HRQoL was assessed using SF-36. Electronic health records were accessed to collect data on demographics, treatments, irAEs and survival. HRQoL data was compared with two norm-based datasets.

**Results:**

Eighty-four metastatic melanoma survivors were eligible and 87% (*N* = 73) completed the SF-36. ICI-related toxicity of any grade occurred in 92% of patients and 43% had experienced a grade 3 or 4 toxicity. Almost half (49%) of the patients required steroids for the treatment of ICI-related toxicity, whilst 14% required treatment with an immunosuppressive agent beyond steroids.

Melanoma survivors had statistically significant lower HRQoL scores with regard to physical, social and physical role functioning and general health compared with the normative population. There was a trend towards inferior scores in patients with previous exposure to ipilimumab compared with those never exposed to ipilimumab.

**Conclusions:**

Our results show that metastatic melanoma survivors have potentially experienced significant ICI-related toxicity and experience significant impairments in specific HRQoL domains. Future service planning is required to meet this population’s unique survivorship needs.

**Electronic supplementary material:**

The online version of this article (10.1007/s00520-019-04818-w) contains supplementary material, which is available to authorized users.

## Background

The introduction of the immune checkpoint inhibitors (ICIs) has transformed the therapeutic arena in metastatic melanoma such that subsets of patients now have the potential to achieve high-quality durable responses and in some cases cure can be achieved. This has translated into an unprecedented number of patients now living with a diagnosis of metastatic melanoma. Oncology clinics worldwide are faced with a new population of patients, the ‘metastatic melanoma survivors’. Many of the principles and applications of cancer survivorship that are common across all survivor populations are relevant to the metastatic melanoma survivors [1, 2]. However, at a time when exciting new therapies are replacing older therapies recognition of tumour-specific and treatment-specific issues that limit the survivor’s return to full physical and psychosocial functioning are necessary to achieve optimal cancer survivor care.

Metastatic melanoma survivors can encounter obstacles in restoration and maintenance in physical domains of health-related quality of life (HRQoL) due to toxicity from ICIs. The immune checkpoints are inhibitory signals that form part of a large network of signalling pathways that act as gatekeepers to the activation of the immune system and regulate the magnitude and duration of the immune response. ICIs are often referred to as ‘the brakes of the immune system’. Removing the ‘brakes’ has the potential to unleash the effectors of the immune system in an unrestrained manner and result in a class of inflammatory adverse events that are unique to ICIs [3, 4]. Frequency of toxicity differs between ICIs. Common toxicities include dermatological toxicity, colitis and hepatitis with rarer toxicities including myocarditis and neurological toxicity [5]. These physical adverse events have the potential to cause significant and persistent morbidity which can occur during therapy but also post-discontinuation. The mainstay of treatment for immune-related adverse events (irAEs) is corticosteroids. Immunomodulatory agents such as infliximab and mycophenolate are utilised in steroid refractory or resistant cases. These therapies can cause toxicities in their own right ranging from issues surrounding glucose tolerance and bone health to viral reactivation and hepatotoxicity. Any survivorship pathway for patients treated with an ICI, which represents the majority of metastatic melanoma survivors, must account for the direct and indirect physical issues irAEs provoke.

The metastatic melanoma survivor will also encounter psychosocial obstacles to restoration of health including uncertainty regarding response to treatment, fear of disease progression or recurrence, negative impact on relationships, work and financial concerns and dealing with unexpected effects of treatment in daily life [6] [7]. The first patient with metastatic melanoma was treated with ipilimumab in a phase 1 trial in 2000. In 2011, ipilimumab was the first ICI to be approved by the FDA and subsequently the EMA for metastatic melanoma. The PD-1 inhibitors nivolumab and pembrolizumab were approved for metastatic melanoma in 2014. This was followed by the approval of the combination of ipilimumab and nivolumab in 2015. Our experience with ICIs in clinical practice is thus relatively limited. Clinical trials do not reflect real-world populations. In a recent report, Donia et al. applied the eligibility criteria from the pivotal phase III trials of ICIs in metastatic melanoma to the Danish metastatic melanoma database and found that 55% of patients would not have met criteria for inclusion [8]. This results in a level of uncertainty regarding aspects of the long-term follow-up of metastatic melanoma survivors. The relative infancy of ICIs in clinical practice also means there are gaps in our knowledge regarding general health issues with examples including fertility and the safety of vaccinations. These uncertainties have the potential to result in significant distress for patients, caregivers and other health professional outside the oncology domain.

Though HRQoL and irAEs have previously been characterised and reported from clinical trials of patients with metastatic melanoma undergoing treatment with ICIs, no report to date has addressed the metastatic melanoma survivors specifically. The aim of this study was to examine melanoma patients’ with metastatic disease, who survived at least 1 year from commencing an ICI and describe toxicity profiles during and after ICI therapy, exposure to immunosuppressive agents and HRQoL in a large single centre in the UK.

## Methods

Between May 2017 and August 2017, all patients who attended the melanoma clinic in the outpatients department at the Royal Marsden NHS foundation trust and who fulfilled eligibility criteria as defined below were invited to fill in the SF-36 questionnaire to assess HRQoL. Patient’s electronic health records were then accessed to collect clinical data including patient demographics, systemic therapies, data regarding prior toxicity (during and after ICI therapy) and treatment of toxicity and survival data.

### Study population

We defined survivors as patients, ≥ 18 years of age, who achieved a durable response to an ICI and had been followed-up for a minimum of 12 months from initiation of ICI without progressive disease, having received at least 1 dose of ipilimumab, nivolumab, pembrolizumab or ipilimumab + nivolumab in the setting of unresectable stage III melanoma or metastatic melanoma as defined by the American joint committee on cancer (AJCC) version 7 staging system [9].

‘Durable response to an ICI’ included (1) patients achieving a response as defined by RECIST criteria (2) patients who had stable disease (SD) as defined by response evaluation criteria in solid tumours (RECIST) for > 24 weeks and (3) patients who were defined as having clinical benefit by their treating physician from ICI in the absence of a RECIST definable response [10, 11]. We adopted this broad definition of response in the knowledge that ICIs can result in unique patterns of response and as a result, patients who derive clinical benefit may not fall within the scope of traditional definitions of response [12].

Patients with isolated areas of disease progression treated with surgery or radiotherapy were included if the remainder of their disease fulfilled our previous definition of response and when radiotherapy or surgery was completed no less than 6 weeks from the date of the SF-36 form being completed. We decided to include these patients as they reflect ‘real-world’ immunotherapy survivors and such patients have the potential to have prolonged survival despite progressive disease [13, 14].

We limited the study to patients who are currently undergoing follow-up in the Melanoma unit of a major UK cancer centre and who were willing and fit to complete the SF-36 questionnaire. Fitness was defined as patients who were physically capable of filling in the form unaided and those who had sufficient capacity to complete the questionnaire.

### Materials and data collection

Following review by the Trusts’ Research and Development department, the study was deemed exempt from full review and approval by a research ethics committee and was considered to fall under ‘Service Evaluation’ (as per HRA guidance) (HRA 2016), given the focus was not on sensitive information and related instead to the treatments received. Aggregated, non-identifiable data only was collated. It was approved by the Trust’s Service Evaluation committee (SE), under the Research and Development department. [15]

Toxicity had been characterised using the immune-related adverse events (irAE) criteria and graded as per Common Terminology Criteria for Adverse Events (CTCAE) version 4.0. Objective responses were classified using the RECIST criteria as described above.

The 36-item short form health survey (SF-36) was used to assess patient-reported HRQoL (Supplementary data). The SF-36 is a validated, self-reported questionnaire covering eight domains of HRQoL: vitality, physical functioning, bodily pain, general health perceptions, physical role functioning, emotional role functioning, social role functioning and mental health [16].

All data regarding toxicity, response and survival was collected up until the date the SF-36 questionnaire was completed to ensure that toxicity and survival data pertained only to the period relevant to the HRQoL data.

### Data synthesis and statistical analysis

Statistical analysis was performed using SPSS version 24. Progression-free survival (PFS) was calculated using the date the last line of systemic therapy was commenced to the date of progressive disease as defined by RECIST criteria. Median PFS were calculated using the Kaplan-Meier method. Differences in SF-36 scores were analysed in pre-planned subgroups categorised based on age, sex, any grade toxicity, grade 3 or 4 toxicity, steroid exposure, ICI type and ipilimumab exposure. Age-based subgroups included the following: adolescents and young adults (AYA) aged 18 to 39 at time of diagnosis, middle-aged aged 40–65 and the elderly aged > 65 years. Dichotomized subgroups (e.g. gender, treatment status) were compared using independent *t* tests. Subgroups with 3 or more categories (e.g. age, ICI type) were compared using an ANOVA test. Norm-based scores from 2 sources were compared with HRQoL outcomes from melanoma survivors: the British office of national statistics (ONS) omnibus survey and the Oxford healthy life survey, both from 1992 [17–19]. Demographic data on norm-based sources is presented in the Supplementary data. Comparison with norm-based data was performed using one-sample *t* test.

## Results

Between January 2011 and August 2016, 481 patients with metastatic melanoma were treated with an ICI. Eighty-four patients (17.5%) met inclusion criteria to be considered part of the survivor population. Seventy-three patients (87%) were willing and fit to complete the SF-36 questionnaire. The 11 patients for whom we do not have HRQoL data include 8 patients who were either uncontactable or declined to partake, 2 patients who were inpatients in hospital during the data collection period (1 was an inpatient for treatment of ICI-induced toxicity, the other admission was unrelated to ICI therapy or melanoma) and 1 patient who was not fit to fill in the SF-36 questionnaire for reasons unrelated to melanoma or its treatment.

### Patient demographics

Patient demographics are presented in Table 1.

**Table 1:** Patient demographics, response & survival

Patient demographics		
	*N* = 84	%
Median age years (range)	65	(22–86)
Male	54	(64)
Female	30	(36)
Histology		
Cutaneous	74	(88)
Mucosal	2	(2)
Unknown	8	(10)
Stage		
IIIC Unresectable	1	(1)
M1a	11	(13)
M1b	14	(17)
M1c	58	(69)
Brain metastases	11	(13)
Lines of therapy		
1	39	(46)
2	30	(36)
≥ 3	15	(18)
Lines of ICI		
1	51	(61)
2	31	(37)
≥ 3	2	(2)
Most recent ICI		
Ipilimumab	16	(19)
Pembrolizumab	31	(37)
Nivolumab	18	(21)
Ipilimumab + nivolumab	12	(14)
Blinded clinical trial^a^	7	(8)
Prior systemic therapy		
BRAF ± MEK inhibitor	15	(18)
Ipilimumab	32	(38)
Pembrolizumab	1	(1)
Nivolumab	1	(1)
Ipilimumab + nivolumab	1	(1)
Chemotherapy	10	(12)
Other	2	(2)
BRAF mutant	30	(36)
LDH < ULN	60	(71)
LDH ≥ ULN	24	(29)
ECOG at start of ICI		
0/1	81	(96)
≥ 2	3	(4)
Autoimmune disease	8	(10)
Responses and survival		
CR	36	(43)
PR	40	(48)
OR	76	(90)
SD	8	(10)
PD^(B)^	13	(15)

*ICI* immune checkpoint inhibitor, *LDH* lactate dehydrogenase, *CR* complete response, *PR* partial response, *OR* objective response, *SD* stable disease, *PD* progressive disease

^a^Arms: ipilimumab + nivolumab, ipilimumab, nivolumab

^b^Progressive disease following initial objective response or stable disease

At the time of analysis, 29.7% of patients were still actively undergoing therapy with an ICI. Thirty-nine percent of patients had stopped treatment due to toxicity. 28.5% of patients had stopped treatment as they had completed therapy as per local guidelines (i.e. 4 cycles of ipilimumab, 2 years of PD-1 inhibitor monotherapy or combination ipilimumab and nivolumab for 4 cycles followed by nivolumab maintenance up to 2 years). Two percent of patients stopped treatment as it was their preference to do so. Patients who have stopped ICI before they completed therapy received a median of 2.1 months (range 0.4–34.6) of systemic therapy.

### Survival

Response and responses presented in Table 1. At the time of analysis, median follow-up was 25 months (95% CI 20.8–29.1). Forty-six percent of patients had been followed-up for 12–24 months, 18% for 25–36 months and 36% for over 36 months since commencing an ICI. Seventy-six patients had a response by RECIST criteria translating into an objective response rate (ORR) of 90%. Eight patients (10%) had SD as best response.

Twelve patients (14%) had experienced isolated sites of progression that had been treated with surgery or radiotherapy. Details of these patients’ best response and treatments are presented in supplementary data**.** Median PFS in this group was 10 months (95% CI 7.7–12.3). Following surgery or radiotherapy, this subgroup has been followed for a median of 13 months (range 4–31) without further intervention for disease progression.

### Toxicity

Rate, class and treatment of irAEs for all patients, patients exposed to ipilimumab and patients solely exposed to PD-1 inhibitors are presented in Table 2.

**Table 2:** Rates of toxicity in the total survivor cohort, in patients who received treatment with ipilimumab either a monotherapy or in combination with a PD-1 inhibitor and patients who received a PD-1 inhibitor

Immune-related adverse events								
	All patients (*N* = 84)^a^				Ipilimumab^b^ (*N* = 59)		PD-1 inhibitor^c^ (*N* = 17)	
	Any grade (%)	Grade 3/4 (%)	Steroid (%)	Immunomodulatory (%)	Any grade	Grade 3 or 4	Any grade	Grade 3 or 4
Total	77 (92)	36 (43)	41 (49)	12 (14)	54 (92)	30 (51)	16 (94)	6 (35)
Colitis	25 (30)	19 (23)	23 (27)	5 (6)	19 (32)	14 (24)	4 (24)	3 (18)
Hepatitis	16 (19)	6 (7)	12 (14)	4 (5)	14 (24)	5 (8)	2 (17)	1 (6)
Msk	23 (27)	4 (5)	5 (6)	2 (2)	17 (29)	4 (7)	5 (29)	0 (0)
Nephritis	3 (4)	3 (4)	3 (4)	1 (1)	2 (3)	2 (3)	1 (6)	1 (6)
Dermatological	59 (70)	10 (12)	26 (31)	0 (0)	36 (61)	7 (19)	14 (82)	2 (12)
Endocrine	26 (31)	6 (7)	6 (7)	0 (0)	21 (36)	5 (8)	5 (29)	1 (6)
Thyroid	17 (20)	0 (0)	0 (0)	0 (0)	13 (22)	0 (0)	4 (24)	0 (0)
Pituitary	6 (7)	2 (2)	2 (2)	0 (0)	6 (10)	2 (2)	0 (0)	0 (0)
Pneumonitis	4 (5)	1 (1)	2 (2)	0 (0)	3 (5)	1 (2)	1 (6)	0 (0)
Neurological	5 (6)	2 (2)	2 (2)	2 (2)	5 (8)	2 (3)	0 (0)	0 (0)

Msk musculoskeletal toxicity arthritis, arthralgia, myalgia

^a^Rates of immune-related adverse events (irAEs) are presented for patients with any grade toxicity and grade 3 or 4 toxicity. Rates of irAEs that required steroids for treatment irrespective of grade are presented (match table) as are rates of irAEs that untimely required treatment with an immunomodulatory agent distinct from steroids (match table)

^b^Rates of irAEs in patients who received ipilimumab during the course of their cancer therapy

^c^Rates of irAEs in patients who received a PD-1 monotherapy during the course of their cancer therapy and were never exposed to ipilimumab

Fourteen percent of patients ultimately required therapies beyond steroids for toxicity including infliximab (*n* = 5), mycophenalate (*n* = 4), vedolizumab (*n* = 1), sulfasalazine (*n* = 1), methotrexate (*n* = 1), eltrombopag (*n* = 1), intravenous immunoglobulins (*n* = 1) and plasmapheresis (*n* = 1).

Twelve patients experienced an irAE having discontinued an ICI. Four such patients developed vitiligo and no other irAE. Characteristics of the remaining 8 patients are presented in Table 3.

**Table 3:** Immune-related adverse events that occurred in patients who discontinued an immune checkpoint inhibitor

Patients who developed irAEs following discontinuation of immune checkpoint inhibitors							
ICI	Reason for discontinuation	Cycles ICI	irAE before discontinuation (grade)	irAE post-discontinuation (grade)	Onset irAE post-discontinuation (months)	Treatment	Best response
Pembrolizumab	irAE	13	Colitis (G2) Rash (G1) Arthralgia (G3)	Myocarditis (G2)	8.1	No intervention required, monitored	SD
Ipilimumab + nivolumab	irAE	2	Aseptic Meningitis (G2) Hepatitis (G3) Colitis (G3)	Arthralgia (G2)	7.4	Steroids	CR
Pembrolizumab	Patient preference	5	Rash (G1) Hepatitis (G2)	Arthralgia (G1)	5.8	Analgesia	PR
Pembrolizumab	irAE	19	Colitis (G3) Arthralgia (G1)	Hepatitis (G3)	3.1	Steroids	SD
Pembrolizumab	irAE	34	Labyrinthitis (G2) Hepatitis (G3) Hypothyroidism (G2) Pruritus (G2)	Arthralgia (G3)	4.9	Steroids Methotrexate	CR
Nivolumab	irAE	3	Colitis (G3)	Rash (G3)	11.0	Topical steroids	CR
Ipilimumab + nivolumab	irAE	1	Rash + Pruritus (G3) Hepatitis (G3) Hypothyroidism (G2) Vitiligo (G1)	Arthralgia (G1)	7.9	Analgesia	CR
Pembrolizumab	irAE	2	Nephritis (G3)	Arthralgia (G2) Rash + Pruritus (G2)	8 12.6	Intra-articular steroids	PR

*irAE* immune-related adverse event, *ICI* immune checkpoint inhibitor, *SD* stable disease, *CR* complete response, *PR* partial response, *G* grade

### HRQoL

Table 4 shows scores for the melanoma survivor’s HRQoL domains as compared with the British ONS omnibus survey and the Oxford healthy life survey norm-based data [17–19]. The melanoma survivors had statistically significant lower scores compared with both norm-based datasets in domains including physical functioning, social functioning, physical role functioning and general health. There was no statistically significant difference between the melanoma survivors scores compared with the norm-based data in domains including mental health and bodily pain. For emotional role, functioning and vitality the melanoma survivors had numerically inferior scores compared with the norm-based data but this only reached statistical significance in comparison with the British ONS survey.

**Table 4:** SF-36 scores for survivor population compared with norm-based data [17–20]

	Patients (mean SF-36)	Oxford (mean SF-36)	95% CI (lower)	95% CI (upper)	*p*^a^	ONS (mean SF-36)	95% CI (lower)	95% CI (upper)	*p*^b^
Physical functioning	74.9	88.4	− 20.3	− 6.5	.000	89.6	− 21.5	− 7.7	.000
Social functioning	80.3	88	− 13.6	− 1.7	.011	89	− 14.6	− 2.7	.005
Role physical	69.1	85.5	− 29.1	− 9.6	.000	84.2	− 24.8	− 5.3	.003
Role emotional	78.5	82.9	− 13.3	4.5	.333	88	− 18.4	− 0.53	.038
Mental health	73.5	73.8	− 4.5	3.9	.890	76.6	− 7.3	1.2	.155
Energy/vitality	55.8	61.1	− 11.2	0.74	.085	64.7	− 14.8	− 2.8	.004
Pain	79.4	81.5	− 8.1	4.04	.505	82.5	− 9.1	3.04	.322
General health	65.3	73.5	− 12.9	− 3.4	.001	74	− 13.4	− 3.9	.001

Mean SF-36 scores for metastatic melanoma survivor population compared with Oxford healthy lifestyle survey normalised data and the British office of national statistics (ONS) form based scores

^a^Comparison of study population with Oxford norm-based data

^b^Comparison of study population and ONS norm-based data

In the subgroup analysis statistically significant differences in physical functioning scores were noted when patients were categorised based on age (Supplementary data)**.** Scores were consistently numerically inferior in elderly patients across all domains which contribute to the physical component score, physical role functioning, general health and bodily pain. These numerical differences resulted in a statistically significant difference in the physical component summary between age groups.

**Fig. 1:**
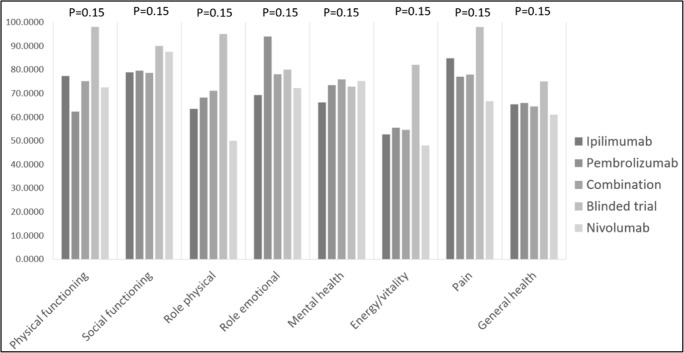
SF-36 scores subcategorised by most recent immune checkpoint inhibitor therapy

**Fig. 2:**
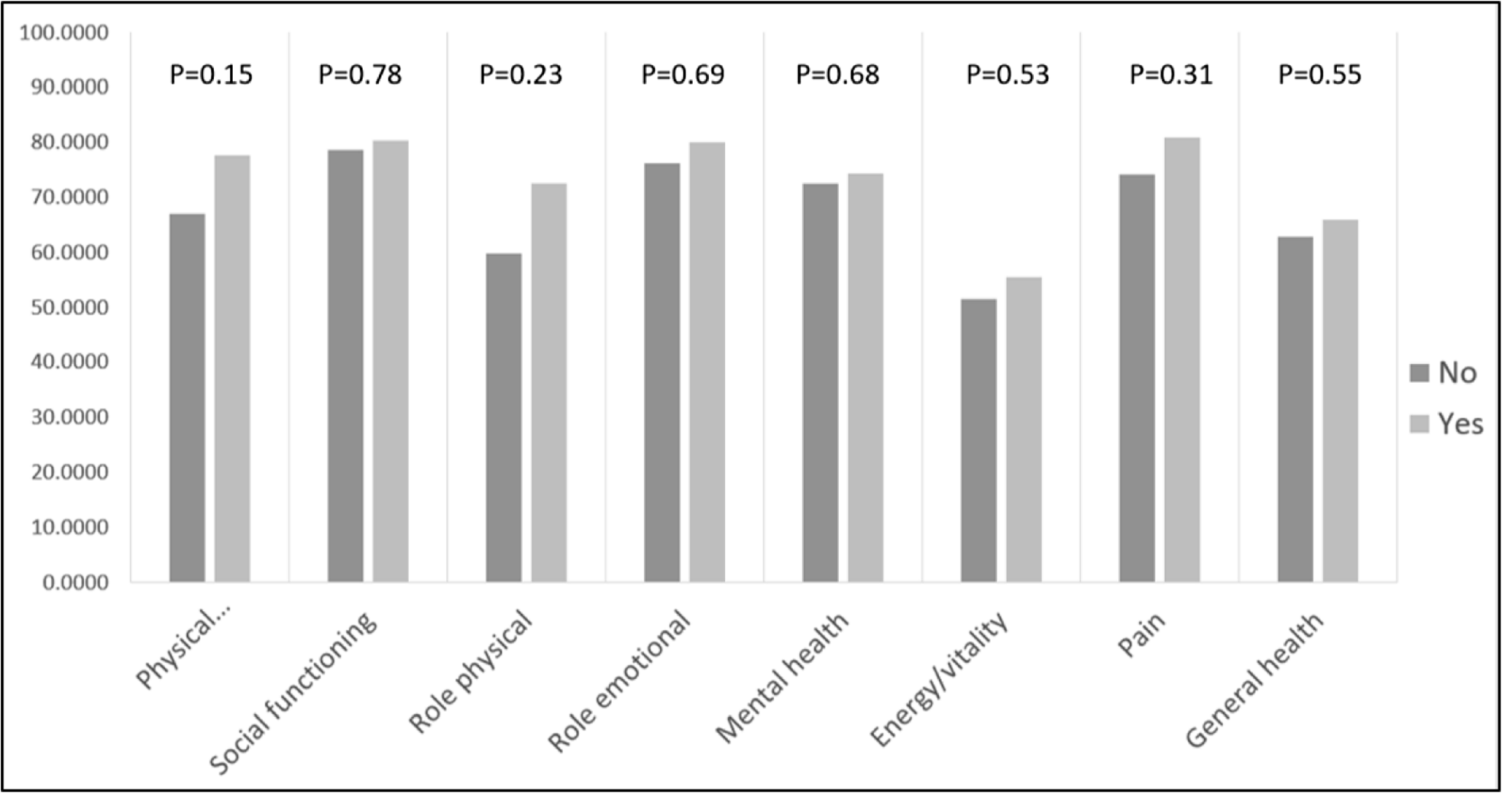
SF-36 scores on patients subcategorised based on exposure to ipilimumab. Yes, exposed to ipilimumab. No, never exposed to ipilimumab

There was considerable variation in scores between patients depending on which ICI they had received **(**Fig. 1**).** There was a trend towards inferior scores in patients with previous exposure to ipilimumab but this did not reach statistical significance **(**Fig. 2**)**. No statistically significant difference in scores was noted in the remainder of the subgroup comparisons including sex, treatment status (on/off treatment), any grade toxicity, grade 3 or 4 toxicity and steroid exposure.

## Discussion

To our knowledge, this is the first report to evaluate HRQoL in metastatic melanoma survivors, showing that survivors have the potential to encounter impairment in both physical and mental HRQoL domains and represent a unique population with specific survivorship needs.

HRQoL has been measured in a number of trials of ICIs in melanoma. To date, four randomised trials have reported patient-reported HRQoL beyond the initial 12 weeks of treatment [21]. In Checkmate 066, a randomised phase III trial comparing nivolumab with chemotherapy, EORTC-QLQ-C30 scores were maintained throughout follow-up for nivolumab up to a maximum of 73 weeks. There were statistically significant improvements in EQ-5D scores from baseline at weeks 7 and 49 and clinically meaningful improvements at weeks 37, 61 and 67 [22]. In Keynote 006, patients treated with pembrolizumab, irrespective of dose, had smaller declines in EORTC-QLQ-C30 scores between baseline and week 12 as compared with ipilimumab (*p* < 0.001). At week 36, for patients who were still on treatment, EORTC-QLQ-C30 scores had improved across all treatment arms and were grossly comparable between treatment groups [23, 24]. In Checkmate 069, no difference was detected between patients who were treated with single agent ipilimumab and combination ipilimumab and nivolumab [25]. In checkmate 067 [26], an initial non-clinically significant decline in patient report HRQoL scores was followed by a return to baseline across all three treatment arms of nivolumab in combination with ipilimumab, ipilimumab monotherapy and nivolumab monotherapy. In each of these four trials, HRQoL data failed to capture patients who discontinued treatment due to toxicity and these was no comparison with norm-based data.

In our survivor cohort, stratification by age resulted in clinically meaningful and statistically significant differences in scores in physical health domains. Elderly patients reported the worst scores. Though recent data would suggest elderly patients do not demonstrate an increased risk of incurring irAEs compared with the remainder of the population, there remains the possibility that they possess reduced physiological reserve and thus toxicity may result in a greater impact on HRQoL [27, 28]. The elderly population is not well represented in previous reports of HRQoL or the clinical trial setting in general [27, 29]. Limitations placed on performance status and organ function mean that elderly patients who do qualify for inclusion may not be representative of the real-world population. Moreover, performance status is a limited tool for evaluating elderly patients and does not encompass important parameters such as frailty comprehensive geriatric assessment may be more effective during and after therapy and should be incorporated into future prospective clinical trials [30].

We found no difference in HRQoL in patients who experienced toxicity and those who experienced no toxicity irrespective of grade and management. This analysis is limited by only six patients in our cohort not experiencing any toxicity; thus, these results should be interpreted with caution. A possible interpretation is that though toxicity is a key contributor to HRQoL, the patient experience has many dimensions that are currently not well captured. We observed a trend towards inferior HRQoL scores in patients exposed to ipilimumab in domains related to physical functioning. This may be related to the fact that these patients experienced more grade 3 or 4 toxicity than patients who never received ipilimumab. In our cohort, hypophysitis (*n* = 6) and neurotoxicity (*n* = 5) only occurred in patients exposed to ipilimumab. Ipilimumab-induced hypophysitis is frequently a chronic condition requiring life-long steroid replacement [31, 32]. Neurological toxicity has the potential to cause significant morbidity [33]. Toxicities of the same grade can affect HRQoL discordantly but this is not reflected in current grading systems.

The SF-36 questionnaire is generic and not cancer specific. This represents a limitation of our study. There are cancer-specific HRQoL questionnaires available (e.g. EORTC-QLQ-C30) that has been favoured for use in clinical trials of ICIs. In terms of being cancer specific, tumour specific, stage specific and treatment specific, all current HRQoL questionnaires available for use in clinic practice are limited. Melanoma-specific modules in HRQoL questionnaires are largely based on long-term sequelae of surgery and though these are contributory to impaired HRQoL, no survey directly deals with the long-term complications of metastatic melanoma or ICI therapy. A study currently underway in Toronto aims to develop a tool based on the Functional assessment in cancer (FACT) tool that is specific to patients undergoing treatment with immunotherapy (NCT02651831) and would thus be more sensitive and relevant to metastatic melanoma survivors. Collection of patient-reported outcomes (PRO) in research and clinical practice is important as it can facilitate patient-centred communication, informed decision making, symptom monitoring and will help to provide patients’ with the best supportive care. In a recent study, Basch et al. demonstrated a 5-month overall survival benefit for patients with metastatic solid tumours undergoing PRO monitoring compared with patients receiving standard care (HR 0.83, 95% CI 0.70–0.99, *p* = 0.04) [34].

Our study is limited by the usage of SF36 questionnaire version 1, newer versions are available. The most recent normative data for the UK population assessed using version 1 was from 1992. This may not be representative of current populations. Whilst the SF-36 questionnaire includes a mental health component, it may be useful in future studies to expand upon this with tools to measure sleep disturbance, distress, anxiety and fear more comprehensively. Our study is limited in that it is cross-sectional in design which hinders the determination of causal associations. There is considerable patient-to-patient variability in factors that can contribute to impairment in HRQoL in metastatic melanoma survivors. These may include severity, timing and chronicity of toxicity as well as quality, timing and durability of response. It is thus not feasible to capture the many potential facets of HRQoL at a single time point. Longitudinal data collection from diagnosis would allow HRQoL to be measured in parallel with key events in treatment and follow-up and would thus be more informative.

## Conclusions

Clinical trial data to date has limited applicability to the survivor population defined in this study owing to limited follow-up time and exclusion of patients who discontinued ICI due to toxicity before a censoring event. Metastatic melanoma survivors have potentially experienced significant irAEs during treatment resulting in chronic conditions, exposure to significant doses of steroids or exposure to other immunomodulatory drugs and thus may encounter long-term sequelae to their cancer and cancer treatment. Our cohort of metastatic melanoma survivors demonstrated significant impairments in physical and mental domains of HRQoL compared with healthy controls. Within our survivorship population, we found that elderly patients were a subgroup that had an increased potential for inferior patient-reported HRQoL outcomes. We strongly support the recognition of the metastatic melanoma survivors as a distinct population that warrant comprehensive longitudinal evaluation with particular focus in area of HRQoL. Future service planning is required to meet these patient’s unique survivorship needs.

## Electronic supplementary material

ESM 1 (DOCX 41 kb)

## Data Availability

Data and material is available upon request from the corresponding author.
