# Supplementary material for: An immunotherapy survivor population: health-related quality of life and toxicity in patients with metastatic melanoma treated with immune checkpoint inhibitors
Source: Support Care Cancer. 2019 May 14;28(2):561–70. doi: 10.1007/s00520-019-04818-w (PMC6954131; doi:10.1007/s00520-019-04818-w)
Supplement: Supplementary file 1 — (DOCX 41 kb) [file 520_2019_4818_MOESM1_ESM.docx]

**Supplementary data Contents**

Table: Patients with isolated sites of progression page 2

Table: British office of national statistics (ONS) omnibus survey and the Oxford healthy life survey norm based data page 3

Figure: SF-36 scores for physical domains for patients subcategorised by age page 4

| **SUPPLEMENTARY DATA** | | | | | |
| --- | --- | --- | --- | --- | --- |
| **PATIENTS WITH ISOLATED SITES OF PROGRESSION** | | | | | |
| **Treatment** | **ICI ongoing** | **Response** | **Time to PD (months)** | **Description** | **Follow-up (months)** |
| Ipilimumab | N | PR | 9 | Multiple isolated relapses surgically resected including; retroperitoneal mass, axillary LN, cervical LN, pelvic LN, omental nodule. | 24 |
| Ipilimumab | N | PR | 10 | Isolated relapse in mesentery resected, new brain metastases treated with cyberknife, ongoing monitoring slowly growing mesenteric nodule. | 31* |
| Ipilimumab | N | PR | 9 | Isolated pulmonary metastases treated with SRS. | 22 |
| Ipilimumab | N | SD | 12 | Resection isolated site soft tissue recurrence. | 16 |
| Nivolumab | N | PR | 31 | Isolated pulmonary metastases treated with SRS. | 15 |
| Pembrolizumab | Y | CR | 11 | Resection isolated site soft tissue recurrence. | 14 |
| Pembrolizumab | N | SD | 6 | Resection isolated LN with PD, Lobectomy for slowly progressive pulmonary metastases. | 4 |
| Nivolumab | N | PR | 10 | Pulmonary relapse treated with resection and SRS. | 13 |
| Pembrolizumab | Y | PR | 13 | Isolated site of PD in pelvic LN treated with SRS. | 12 |
| Pembrolizumab | Y | PR | 5 | Isolated site of PD in acetabulum treated with SRS. | 10 |
| Ipilimumab | N | CR | 37 | Resection isolated liver lesion | 5 |
| Pembrolizumab | Y | PR | 22 | Slow PD in isolated vertebral lesion resected | 4 |
| Patients who had isolated sites of progression post ICI treatment that were then treated with radiotherapy or surgery. ICI Immune checkpoint inhibitor, PD Progressive disease, PR Partial response, CR Complete response, SD Stable disease, LN Lymph node, SRS Stereotactic radiosurgery, *Time from last intervention for isolated site of progression. | | | | | |

| **Supplementary Data** | | |
| --- | --- | --- |
| **British office of national statistics (ONS) omnibus survey and the Oxford healthy life survey norm based data[18]** | | |
|  | Oxford  (n=9332) | ONS  (n=2051) |
| Age range | | |
| < 65 | 9332 (100%) | 1553 (76%) |
| ≥ 65 | 0 (0%) | 498 (24%) |
| Gender | | |
| Male | 4229 (45%) | 929 (45%) |
| Female | 5103 (55%) | 1122 (55%) |
|  | | |

**Figure Supplementary data:** SF-36 scores for physical domains for patients subcategorised by age


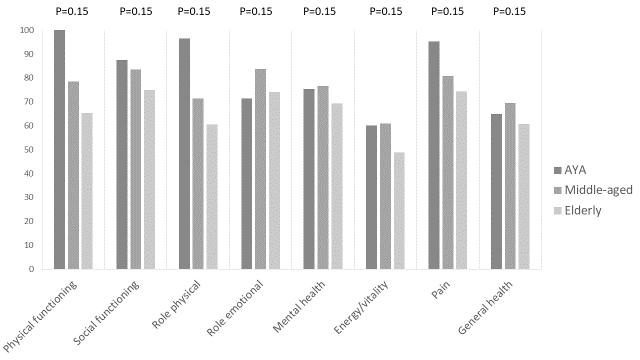


**SF-36 scores for physical domains for patients subcategorised by age. AYA adolescents and young adults**
